# Hippocampal and Hippocampal-Subfield Volumes From Early-Onset Major Depression and Bipolar Disorder to Cognitive Decline

**DOI:** 10.3389/fnagi.2021.626974

**Published:** 2021-04-21

**Authors:** Niels Hansen, Aditya Singh, Claudia Bartels, Frederic Brosseron, Katharina Buerger, Arda C. Cetindag, Laura Dobisch, Peter Dechent, Birgit B. Ertl-Wagner, Klaus Fliessbach, John D. Haynes, Michael T. Heneka, Daniel Janowitz, Ingo Kilimann, Christoph Laske, Coraline D. Metzger, Matthias H. Munk, Oliver Peters, Josef Priller, Nina Roy, Klaus Scheffler, Anja Schneider, Annika Spottke, Eike J. Spruth, Stefan Teipel, Maike Tscheuschler, Ruth Vukovich, Jens Wiltfang, Emrah Duezel, Frank Jessen, Roberto Goya-Maldonado

**Affiliations:** ^1^Department of Psychiatry and Psychotherapy, Göttingen, Germany; ^2^Laboratory of Systems Neuroscience and Imaging in Psychiatry, University Medical Center Göttingen, Göttingen, Germany; ^3^German Center for Neurodegenerative Diseases (DZNE), Bonn, Germany; ^4^Department for Neurodegenerative Diseases and Geriatric Psychiatry, University Hospital Bonn, Bonn, Germany; ^5^German Center for Neurodegenerative Diseases (DZNE, Munich), Munich, Germany; ^6^Institute for Stroke and Dementia Research (ISD), University Hospital, LMU Munich, Munich, Germany; ^7^Berlin Institute of Health, Institute of Psychiatry and Psychotherapy, Charité—Universitätsmedizin Berlin, Corporate Member of Freie Universität Berlin, Humboldt-Universität zu Berlin, Berlin, Germany; ^8^German Center for Neurodegenerative Diseases (DZNE), Berlin, Germany; ^9^German Center for Neurodegenerative Diseases (DZNE), Magdeburg, Germany; ^10^MR-Research in Neurology and Psychiatry, University Medical Center Göttingen, Göttingen, Germany; ^11^Institute for Clinical Radiology, Ludwig-Maximilians-University, Munich, Germany; ^12^Bernstein Center for Computational Neuroscience, Charité—Universitätsmedizin, Berlin, Germany; ^13^German Center for Neurodegenerative Diseases (DZNE), Rostock, Germany; ^14^Department of Psychosomatic Medicine, Rostock University Medical Center, Rostock, Germany; ^15^German Center for Neurodegenerative Diseases (DZNE), Tübingen, Germany; ^16^Section for Dementia Research, Hertie Institute for Clinical Brain Research, Tübingen, Germany; ^17^Department of Psychiatry and Psychotherapy, University of Tübingen, Tübingen, Germany; ^18^Institute of Cognitive Neurology and Dementia Research (IKND), Otto-von-Guericke University, Magdeburg, Germany; ^19^Department of Psychiatry and Psychotherapy, Otto-von-Guericke University, Magdeburg, Germany; ^20^Department of Psychiatry and Psychotherapy, Berlin, Germany; ^21^Department for Biomedical Magnetic Resonance, University of Tübingen, Tübingen, Germany; ^22^Department of Neurology, University of Bonn, Bonn, Germany; ^23^Department of Psychiatry and Psychotherapy, University of Cologne, Medical Faculty, Cologne, Germany; ^24^German Center for Neurodegenerative Diseases (DZNE), Göttingen, Germany; ^25^Neurosciences and Signaling Group, Department of Medical Sciences, Institute of Biomedicine (iBiMED), University of Aveiro, Aveiro, Portugal; ^26^Excellence Cluster on Cellular Stress Responses in Aging-Associated Diseases (CECAD), University of Cologne, Köln, Germany

**Keywords:** Alzheimer's disease, cognitive impairment, early-onset depression, hippocampus, hippocampal subfields, MRI volumetry

## Abstract

**Background:** The hippocampus and its subfields (HippSub) are reported to be diminished in patients with Alzheimer's disease (AD), bipolar disorder (BD), and major depressive disorder (MDD). We examined these groups vs healthy controls (HC) to reveal HippSub alterations between diseases.

**Methods:** We segmented 3T-MRI T2-weighted hippocampal images of 67 HC, 58 BD, and MDD patients from the AFFDIS study and 137 patients from the DELCODE study assessing cognitive decline, including subjective cognitive decline (SCD), amnestic mild cognitive impairment (aMCI), and AD, via Free Surfer 6.0 to compare volumes across groups.

**Results:** Groups differed significantly in several HippSub volumes, particularly between patients with AD and mood disorders. In comparison to HC, significant lower volumes appear in aMCI and AD groups in specific subfields. Smaller volumes in the left presubiculum are detected in aMCI and AD patients, differing from the BD group. A significant linear regression is seen between left hippocampus volume and duration since the first depressive episode.

**Conclusions:** HippSub volume alterations were observed in AD, but not in early-onset MDD and BD, reinforcing the notion of different neural mechanisms in hippocampal degeneration. Moreover, duration since the first depressive episode was a relevant factor explaining the lower left hippocampal volumes present in groups.

## Introduction

The human hippocampus is known as a brain structure pivotal for memory formation. It is the plasticity of the hippocampus to form memory that makes it particularly vulnerable to damage and volume reduction. In Alzheimer's disease (AD), hippocampal volume is reduced due to neurodegeneration as evidenced in brain MRIs of specific hippocampal subfields (HippSub). A variety of human studies have reported that specific HippSubs such as the cornu ammonis 1–3 (CA1–3), presubiculum or subiculum are more prone to neurodegenerative processes than others (Hanseeuw et al., 2011; La Joie et al., 2013; Carlesimo et al., 2015; de Flores et al., 2015). The degeneration pattern may depend on the AD stage, as indicated by cognitive performance, varying from subjective cognitive decline (SCD) to dementia. HippSub fields are suitable biological imaging markers of early stages of AD, as the presubiculum-subiculum complex (Carlesimo et al., 2015; Jacobs et al., 2020), CA2–3 (Hanseeuw et al., 2011), or CA1 region (de Flores et al., 2015) are often atrophied. Supporting this idea, recent work indicates that lower subicular volumes in patients with memory impairment are related to the grade of ß-amyloid depositions independent of the presence of neurodegeneration assessed by fluorescence desoxyglucose positron emission tomography (FDG PET) (Filho et al., 2021). More broadly, another study confirmed the association of ß-amyloid deposition in conjunction with higher iron content in the medial temporal lobe and subjects' age (even in cognitively unimpaired subjects) in terms of specific HippSub volume decreases, i.e., in the subiculum, CA1/2, CA3/dentate gyrus (DG) subregions (Foster et al., 2020). ß-amyloid accumulation is a key underlying mechanism in the loss of hippocampal volume across the spectrum of cognitive impairment in preclinical and clinical AD. Another study suggest that both reduced cerebrospinal fluid (CSF) ß-amyloid 1-42 and elevated CSF tau levels are seen in AD patients who exhibit smaller subiculum volumes (Tardif et al., 2018). This evidence suggests that both tau-based neurodegeneration and ß-amyloid pathology are crucial for HippSub volume loss in patients with AD. Other mechanisms underlying the loss of hippocampal volume might be polygenic, as a higher polygenic risk score for AD was observed in cognitively normal patients in a study by Foo (Foo et al., 2020), possibly depicting preclinical AD. Protective mechanisms might also play a role, such as carrying the TREML2 rs3747742-C polymorphism, which seem related to higher CA1 volumes in cognitively normal subjects (Wang et al., 2020). The interrelationship between depression and AD is a well-replicated finding (Heser et al., 2013; Donovan et al., 2018). It remains unclear whether depression is a relevant risk factor for AD (Enache et al., 2011), or if depression is an early manifestation thereof (Singh-Manoux et al., 2017). Furthermore, there is recent evidence that a decrease in hippocampal volume and functional connectivity is an important feature of major depressive disorder (MDD) associated with cognitive impairment (Genzel et al., 2015; Schmaal et al., 2016). Thus, it is of major interest to compare HippSub volumes which might give us hints about common underlying mechanisms in affective disorders and AD. In depressive disorders, diverse mechanisms such as the number of depressive episodes, stressful life events, oxidative stress, childhood physical, or sexual abuse or metabolic changes are potential underlying mechanisms of lower HippSub volumes such as CA1 or dentate gyrus (DG) or fimbria (Treadway et al., 2015; Elvsåshagen et al., 2016; Xu et al., 2018; Weissman et al., 2020; Yuan et al., 2020). These studies depict that in depression, the mechanisms of hippocampal volume loss seem to be even broader than in hippocampal degeneration due to AD's spectrum. HippSub loss does not just concern unipolar depression; it is also present in bipolar disorder (BD); the pattern of subfield loss was considerably more extensive than in controls in a recent multicentric study with 1,472 BD patients (Haukvik et al., 2020). Another recent study indicated one possible common pathogenic mechanism between BD and AD (Berridge, 2013), which is why we added a BD group in our study. BD could could result in a HippSub-specific fingerprint like reduced volume in the CA1 (Cao et al., 2017; Haukvik et al., 2020), cornu ammonis 4 (CA4) (Cao et al., 2017; Haukvik et al., 2020), the granule cell layer (GCL) (Cao et al., 2017; Haukvik et al., 2020), molecular layer (ML) (Cao et al., 2017; Haukvik et al., 2020), subiculum (Sub) (Cao et al., 2017; Haukvik et al., 2020), hippocampal amygdala transition area (Haukvik et al., 2020) and tail (Cao et al., 2017; Haukvik et al., 2020) depending on the duration and type of BD (Cao et al., 2017), but also on antipsychotic and antiepileptic drug history (Haukvik et al., 2020). On the other hand, it has been suggested that depressive symptoms might reduce age-related hippocampal atrophy and result in larger Sub and CA1 subfields (Szymkowicz et al., 2017). However, most studies showed smaller hippocampal volumes due to ongoing depressive symptoms, thus the controversy about how depression's duration relates to HippSub volumes. The aforementioned studies show that the mechanism of hippocampal volume loss might differ even in two distinct affective disorders and AD and that it is not fully understood. However, we wondered whether there might be a similar pattern of HippSub loss in some HippSubs implying similar mechanisms of degeneration.

In the current investigation, we thus aimed [a] to analyze HippSub volumes and hippocampal volumes between cohorts with cognitive impairment, early-onset major depression and BD, and [b] to identify potential disorder-specific alterations and any shared trajectories of hippocampal volume decrease in the hippocampus and HippSub in SCD, aMCI, AD, BD, and MDD groups. Our study covers the spectrum ranging from subjective complaints (SCD) to amnestic mild cognitive impairment (aMCI) and AD. SCD patients do not reveal objective cognitive impairment. Therefore, it is worth seeking novel biomarker tools such as hippocampus and HippSub imaging to diagnose early AD more accurately. In addition, we are looking for molecular markers in the CSF such as ß-amyloid and tau protein to detect any underlying pathomechanism for HippSub in AD; a recent study by Tardif (Tardif et al., 2018) proved a relevant relationship between HippSub decline and ß-amyloid and tau-based neuropathology in AD. Our study does not focus on specific HippSubs, as there is controversy about which HippSubs are reduced among different diseases. The intersection between lower HippSub volumes and various diseases associated with cognitive dysfunction is inconsistent in studies of AD's spectrum (Hanseeuw et al., 2011; La Joie et al., 2013; Carlesimo et al., 2015; de Flores et al., 2015; Cao et al., 2017; Szymkowicz et al., 2017; Jacobs et al., 2020), MDD (Treadway et al., 2015; Elvsåshagen et al., 2016; Xu et al., 2018; Weissman et al., 2020; Yuan et al., 2020), and BD (Cao et al., 2017; Haukvik et al., 2020). Therefore, we plan to take a more exploratory look at the volumes of various HippSubs. Furthermore, we aimed to discover whether specific factors show a relevant impact on our HippSub and hippocampal volumes in certain disease groups; i.e., sex, age, disease duration, age at condition onset, number of depressive episodes, duration since first depression, and intracranial volume. In addition, we expected to uncover potential relationships not yet investigated between hippocampal volume and HippSub volumes and duration since the first occurrence of a depressive episode between all groups that might be clinically relevant and thus support the relevance of very early, effective treatment to impede further hippocampal degeneration that might accompany disease progression. By analyzing early-onset depression and BD patients, we will demonstrate a wide spectrum of time duration in years between the first episode of depression and hippocampal and HippSub volumes to answer how a lifetime's duration of suffering intermittent depressive and no depressive episodes since the first one's occurrence relates to hippocampus volumetry. Analyzing hippocampal volumes in addition to the HippSubs is an important endeavor, as they involve functional aspects of memory such as pattern separation and recognition in AD (Rizzolo et al., 2021), stress sensitization (Weissman et al., 2020), as does the number of depressive episodes in prior life (Videbech and Ravnkilde, 2004).

## Methods

### Participants

We compared data of two independent cohorts from 137 patients of the DELCODE study and 58 patients of the AFFDIS study in this retrospective investigation. The German DELCODE [**D**eutsches Zentrum für Neurodegenerative **E**rkrankungen (DZNE, German Center for Neurodegenerative Diseases) **L**ongitudinal **CO**gnitive impairment and **De**mentia] is assessing cognitive decline and dementia in an ongoing, memory clinic-based, observational, longitudinal, multicentric study (Jessen et al., 2018). The AFFDIS study investigated differential neural correlates in **AFF**ective **DIS**orders (AFFDIS) and medication-related changes from 2015 to 2017. For a detailed description of the DELCODE study design and study population, please see Jessen et al. (2018). In short, participants from the DELCODE cohort were grouped into SCD (*n* = 32; mean age: 72 ± 6.2 years, age range: 60–89 years), amnestic mild cognitive impairment (aMCI) (*n* = 63; mean age: 72.5 ± 5.9 years, age range: 62–88 years), and AD (*n* = 42; mean age: 72.9 ± 6.9 years, age range: 61–87 years). The AD patients were selected according to McKhann's criteria (McKhann et al., 2011). Probable AD is diagnosed according to McKhann's criteria (McKhann et al., 2011) when the following deficits and other alternative causes have been excluded: a gradual, not acute onset of symptoms, worsening cognition resulting in dementia with a prominent amnestic presentation of cognitive dysfunction, difficulty finding words and solving problems, defective spatial cognition, impaired reasoning, or judgement. We randomly selected the patients from the DELCODE cohort for comparable size between study cohorts (AFFDIS, DELCODE) and their subgroups. Participants were classified as having SCD in case of self-reported subjective cognitive decline and a neuropsychological test achievement superior than −1.5 standard deviation (SD) on each subtest of the Consortium to Establish a Registry for Alzheimer's Disease (CERAD) test battery (according to normative data adapted for age, education and sex) (Jessen et al., 2014, 2018, 2020). According to research criteria (Jessen et al., 2018), participants with aMCI were defined as those whose neuropsychological performance was below −1.5 SD in the delayed recall test of the CERAD word list, which is indicative of episodic memory. For the HC group (*n* = 67, age: 54.0 ± 16.7 years, age range: 19–78 years) from the DELCODE study, the same test criteria for SCD were applied, but subjective cognitive concerns were absent. In a subgroup of patients with cognitive impairment in the DELCODE study [21/32 (66%) SCD, 46/63 (73%) aMCI, and 19/42 (45%) AD patients] cerebrospinal fluid (CSF) biomarkers were assessed. As part of the DELCODE protocol, Tau-protein, phosphorylated 181 Tau-protein (pTau181), ß-Amyloid 42, ß-Amyloid 40, and the ratio of ß-Amyloid 42/40 were analyzed in cerebrospinal fluid (CSF) with cut-off values for AD's molecular markers established at the University Hospital in Bonn as previously described (Jessen et al., 2018). AD's molecular signature (AD pathology+) was present if Aß42 or the Aß42/Aß40 ratio in CSF was reduced and Tau protein or pTau181 were elevated in CSF in line with Jack's criteria for biological AD (Jack et al., 2018).

Major exclusion criteria were significant sensory impairment, major or neurological psychiatric disorder, current major depressive episode, malignant disease, cerebral ischemia, Vitamin B12 deficiency, and any unstable medical condition. A medical history derived from the participant's and caregiver's self-reports was collected and covered depression history (e.g., age of depression onset, number of previous mood episodes, if applicable). In the AFFDIS cohort, participants with affective disorders were diagnosed with BD (*n* = 28, age: 54.0 ± 16.7 years, and age range: 26–63 years) and MDD (*n* = 30, age: 38.2 ± 15.9 years, and age range: 19–65 years), according to the DSM-5 criteria, and were assessed by the Beck Depression Inventory-II (BDI-II), while HC participants were evaluated by the Symptom Checklist-90-R (SCL-90-R) to ensure the absence of psychopathological symptoms. By pooling HC from the two cohorts (DELCODE *n* = 32, AFFDIS *n* = 35), the HC group consisted of 67 participants in total. Informed consent was received from all participants. Approval was obtained for DELCODE [ethics committee of the University Hospital Bonn and subsequent local ethics committee's of the participating centers of Berlin (Charité-Universitätsmedizin Berlin), Göttingen (University Medical Center of Göttingen), Cologne (University Hospital Cologne), Magdeburg (Otto-von-Guericke University Magdeburg), Munich (LMU Munich), Rostock (University Medical Center of Rostock), and Tübingen (University of Tübingen)] and AFFDIS (ethics committee of the University Medical Center of Göttingen) from our local ethics committee and for DELCODE from the executive board of the DZNE in Bonn, Germany. The study was in agreement with the guidelines of the Declaration of Helsinki.

### Neuroimaging

**Figure 1:**
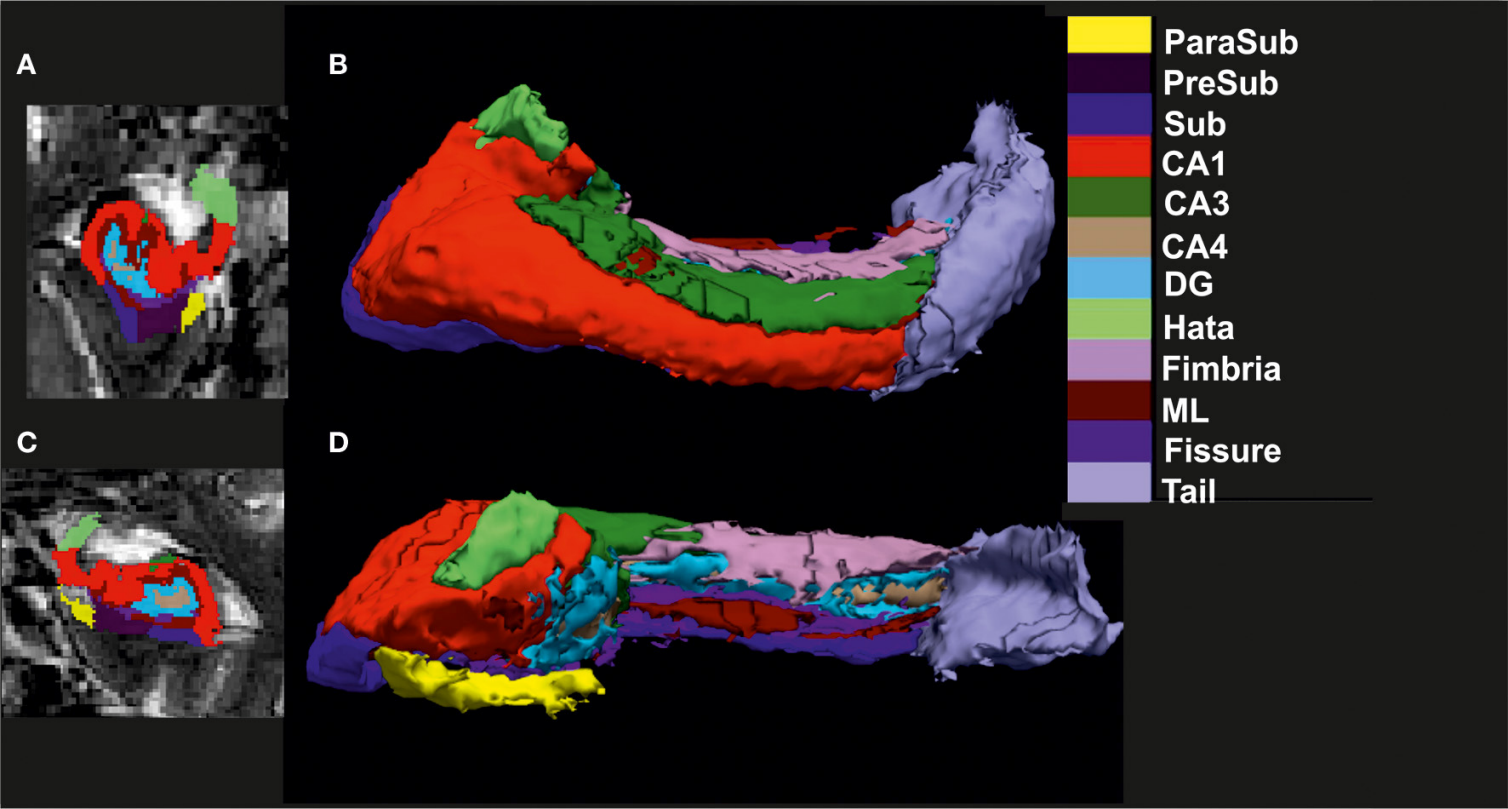
Visualization of hippocampal subfield segmentation. **(A)** Left hippocampal subfields (HippSub) presented in a coronal MRI section, **(B)** Left HippSub illustrated in a 3D reconstruction, **(C)** Right HippSub presented in a coronal MRI section, and **(D)** Right HippSub illustrated in a 3D reconstruction. HippSub color code is on the right side of the figure. CA1/3/4, cornu ammonis 1/3/4; DG, granule cell layer-molecular layer of the dentate gyrus; Hata, hippocampus-amygdala transition area; ML, molecular layer; ParaSub, Parasubiculum; PreSub, Presubiculum; Sub, Subiculum.

We used whole-brain T1-weighted images (1 mm isotropic) and high-resolution T2-weighted images (0.5 × 0.5 × 1.5mm^3^) spanning the hippocampus to segment it into its constituent substructures. These structural images were acquired using 3T MRI Siemens scanner systems [TIM Trio and Verio systems, Skyra, and Prisma system, both the DELCODE and AFFDIS cohorts. We used the already established and reliable method, corroborated by longitudinal studies (Brown et al., 2020; Garimella et al., 2020; Xu et al., 2020), of FreeSurfer (Version 6.0, software: http://surfer.nmr.mgh.harvard.edu/) to segment the whole brain T1-weighted structural images using the default standard recon-all processing stream (Dale et al., 1999; Fischl et al., 1999). This step usually takes about 7–10 h for each subject image, and outputs the segmentation results from both cortical and subcortical structures. Standard preprocessing comprises brain extraction, B1 bias field correction, segmentation of gray as well as white matter, reconstruction of gray matter–white matter boundary and pial surfaces, labeling of regions in both the cortex and subcortex, and non-linearly co-registering the individual T1's cortical surface to a spherical atlas to allow comparison across subjects. To obtain HippSub segmentation, we employed the higher-resolution T2-weighted scans using the revised module available in FreeSurfer 6.0 (Iglesias et al., 2015; Whelan et al., 2016). The step takes ~45 min for each subject's hippocampal segmentation and provides a label for the following subregions: hippocampal tail, subiculum (Sub), CA1, fissure, presubiculum (PreSub), parasubiculum (ParaSub), molecular layer (ML), granule cell layer-molecular layer of the DG, CA3, cornu ammonis 4 (CA4), fimbria, and hippocampus-amygdala transition area (Hata) region in both hemispheres. After this, we used automated scripts (courtesy of P. Saemann of the ENIGMA consortium [https://enigma.ini.usc.edu]) to extract the HippSub volumes of each hemisphere for further statistical analysis. Finally, we created 2D and 3D (Figure 1) renderings to perform careful quality check (QC) to ensure correct segmentation of all cases before running statistical analysis. Cases of poorly segmented hippocampus or HippSub were absent.

### Statistical Analysis

We performed ANOVA to detect differences between groups and controls in relevant variables such as sex, age, disease duration, age at condition onset, number of depressive episodes, duration since first depression, and intracranial volume (eTIV). We examined the potential contribution of covariates (age, age at condition onset, and eTIV) to the HippSub volumes as they showed significant group differences. Only those covariates exhibiting relevant group differences among all patients were regarded as significant covariates in our HippSub analysis. To investigate volume differences between all groups, we analyzed HippSub volumes from FreeSurfer using ANOVA with group as a factor (SCD, aMCI, AD, BD, and MDD) with and without HC and with covariates age and eTIV. An additional one-way ANOVA was performed only with the cognitive-decline-groups as factor with or without CSF pathology suggestive of Alzheimer's disease (SCD, aMCI, AD, SCD-CSF pathology+, aMCI-CSF pathology+, and AD-CSF pathology+). A further ANOVA was performed for AFFDIS patient groups and their AFFDIS control group with eTIV as covariate. To investigate the potential impact of time since first depressive episode on volume reduction, we ran a linear regression analysis in all patient groups that had history of depression. The length of time since the first depressive episode is defined as the cumulative amount of time someone had been depressed including transient time periods with no depression in their lifetime before hippocampal volume was assessed. Statistical analysis was performed via SPSS (Version 25, IBM Inc., Chicago, Illinois, USA). Graphs were constructed by Sigma Plot (Version 11, Sigma Plot, USA). Statistical analyses were two-sided with a *p*-level of significance ≤ 0.05, including, if applicable, LSD *post-hoc* tests including Bonferroni correction.

## Results

### Baseline Characteristics of Groups

We pooled HC (*n* = 67) from the AFFDIS cohort (*n* = 35) and DELCODE cohort (*n* = 32) to serve as a reference for potential effects of age-related differences in hippocampus and HippSub volumes. Clinic and demographic data of study participants (*n* = 195) are presented in Table 1, showing sex, age, onset age of depressive episodes, number of depressive episodes, age at onset of condition, and duration since first depression compared across all groups (HC, SCD, aMCI, AD, BD, and MDD). Past depressive episodes were identified in 7/32 (22%) of SCD, in 5/63 (7.9%) of aMCI and in 4/42 (9.5%) of AD patients. The BP and MDD patients revealed a moderate degree of current depressive mood a s indexed by BDI-II (BDI-II scores: BD: 19 ± 12.8; MDD: 25 ± 11.3). Age (*F* = 68.9, *p* < 0.005), disease condition's onset age (*F* = 90.7, *p* < 0.005), and the onset age of depressive episodes (*F* = 4.3, *p* < 0.005) and the duration of depression (*F* = 4.4, *p* < 0.005) differed significantly between groups, whereas sex and number of depressive episodes did not. The eTIV differed significantly between groups (*F* = 4.98, *p* < 0.0005). In *post-hoc* analysis, only SCD and HC differed significantly from BD and MDD patients (*post-hoc* test: *p* < 0.05), while the other groups did not (LSD *post-hoc* test: *p* > 0.05). However, when comparing the HC in the AFFDIS cohort only with BD and MDD patients in eTIV volume, we detected no significant differences (LSD *post-hoc* test: *p* > 0.05). Thus, the eTIV difference was driven by the SCD group compared with BD and MDD patients. Overall, age and eTIV showed relevant group differences among all patients and were considered as relevant covariates for our HippSub analysis as well as linear regression of hippocampus and HippSub volumes in patients with and without controls.

**Table 1:** Demographic and clinical information of patient and control groups.

		**DELCODE cohort**			**AFFDIS cohort**		**Statistics**
	**SCD**	**MCI**	**AD**	**BD**	**MDD**	**HC (HCDELCODE, HCAFFDIS)**	***F*, *p***
Number of subjects/patients	*n* = 32	*n* = 63	*n* = 42	*n* = 28	*n* = 30	*n* = 67 (32, 35)	
Sex (females/ males)	15/17	34/29	18/24	17/11	16/14	26/ 41 (22/ 10, 19/ 16)	68.9, 0.371 (0.342)
Age (y)	72 ± 6.2	72.5 ± 5.9	72.9 ± 6.9	44 ± 9.7	38.2 ± 15.9	54.0 ± 16.7 (67.4 ± 4.3, 41.4 ± 14.3)	1.082, <0.005 (<0.0005)
Age at disease onset (y)	56.7 ± 6.9	57.8 ± 5.0	59.5 ± 7.9	26.4 ± 9.8	28 ± 15.6	na	102.6, <0.0005
Onset of depressive episodes (y)	46.9 ± 17.7	36.4 ± 22.2	49.75 ± 15.9	25.7 ± 11.1	28.7 ± 15.9	na	4.81, <0.0005
Number of depressive episodes	2.7 ± 3.3	2.25 ± 1.2	2 ± 1.15	6.6 ± 5.5	4.8 ± 4.4	na	2.07, 0.095
Duration of depression (y)	21 ± 18.7	33.8 ± 26.4	17.5 ± 15.5	5 ± 12.75	9.4 ± 9.3	na	4.42, <0.005

*AD, Alzheimer's disease dementia; BD, bipolar disorders; HC, healthy controls; HCDELCODE, healthy controls DELCODE; HCAFFDIS, healthy controls AFFDIS; MCI, mild cognitive impairment; MDD, major depressive disorder; na, not available; SCD, subjective cognitive decline; y, years; Mean ± standard deviation*.

### Comparison of Hippocampal Subfield Volumes Between Cognitive Decline and Affective Disease Groups Without Controls

**Figure 2:**
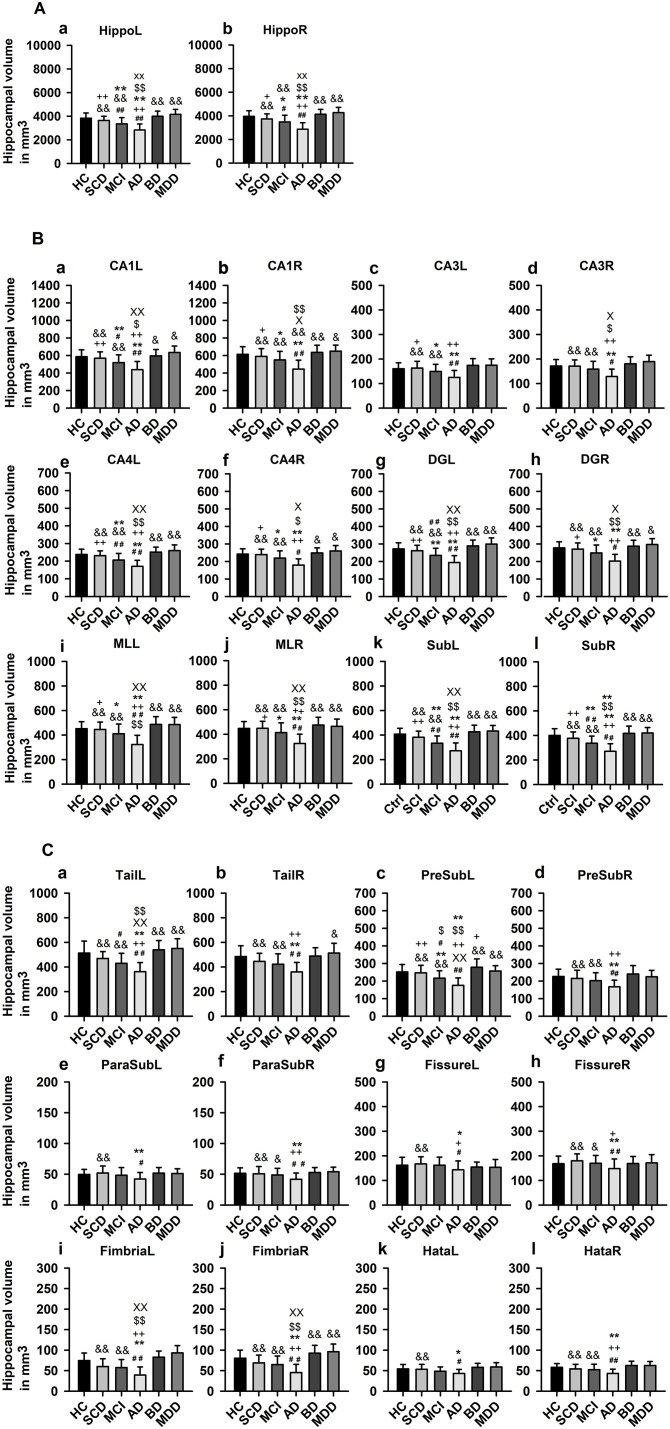
Hippocampal subfield volumes across groups. **(A)** Whole hippocampus volumes compared in each hemisphere, **(B)** Hippocampal subfield (HippSub) volumes, including the CA1, CA3, CA4, DG, ML, and Sub as part of the hippocampus, compared in each hemisphere, and **(C)** Additional HippSub volumes including tail, PreSub, ParaSub, fissure, fimbria, Hata, compared in each hemisphere. Results refer to LSD *post-hoc t*-tests (two-sided) with Bonferroni correction between each condition. The significance level is indicated by different symbols: ^##^*p* < 0.005 vs. HC, **p < 0.005 vs. SCD, ^++^*p* < 0.005 vs. aMCI, ^&^*p* < 0.005 vs. AD, ^$$^*p* < 0.005 vs. BD, ^xx^*p* < 0.005 vs. MDD, *p < 0.05 vs. SCD, ^+^*p* < 0.05 vs. aMCI, ^&^*p* < 0.05 vs. AD, ^$^*p* < 0.05 vs. BD, ^x^*p* < 0.05 vs. MDD. AD, Alzheimer's disease; BD, bipolar disorder; CA1/3/4, cornu ammonis 1/3/4; HC, healthy controls; DG, granule cell layer-molecular layer of the dentate gyrus; Hata, hippocampus-amygdala transition area; L, left; aMCI, amnestic mild cognitive impairment; MDD, major depressive disorder; ML, molecular layer; ParaSub, Parasubiculum; PreSub, Presubiculum; R, right; SCD, subjective cognitive decline; Sub, Subiculum.

ANOVA revealed a significant difference (*F* = 2.24, *p* < 0.0005, see Table 2 for neuroimaging data of patients and controls) in hippocampus and HippSub volumes between all groups including cognitive decline (SCD, aMCI, and AD) and early-onset mood conditions (MDD and BD). The hippocampus in both hemispheres exhibited smaller volumes in AD patients, but not in MDD and BD patients (LSD *post-hoc* test: *p* < 0.0005; Figure 2A). Bilateral CA1, CA4, DG, ML, Sub, fimbria, and left tail revealed the same pattern of a diminished volume in AD, but not in MDD and BD groups (LSD *post-hoc* test: *p* < 0.05, Figures 2B,C). Significantly lower volumes in the left PreSub were observed in aMCI and AD patients when compared to BD (LSD *post-hoc* test: *p* < 0.005, Figures 2B,C). No differences between hippocampal volumes in AD vs. BD or MDD patients were identified in bilateral CA3, ParaSub, fissure, hata, and right PreSub regions (Figures 2B,C).

**Table 2:** Neuroimaging data of patient and control groups.

	**DELCODE cohort**			**AFFDIS cohort**		
	**SCD**	**aMCI**	**AD**	**BD**	**MDD**	**HC**
eTIV	1, 412, 486, 223, 372	1, 490, 137, 267, 502	1, 468, 571, 141, 138	1, 575, 000, 188, 277	1, 575, 667, 134, 976	141, 908, 194, 631
**Right side**						
Whole Hippocampus	2, 921, 361	2, 714, 436	2, 205, 426	3, 179, 339	3, 229, 307	3, 051, 351
CA1	590, 88	549, 99	445, 102	636, 82	649, 70	613, 86
CA3	171, 25	159, 32	128, 30	180, 29	189, 26	171, 26
CA4	239, 31	219, 41	179, 35	249, 29	260, 31	242, 30
DG	270, 35	248, 45	202, 39	287, 34	296, 34	277, 35
Fimbria	68, 19	65, 21	45, 20	93, 19	96, 18	80, 27
Fissure	180, 29	170, 32	148, 39	168, 29	171, 34	168, 31
Hata	54, 11	52, 14	43, 10	63, 10	63, 10	58, 9
Molecular Layer	270, 35	414, 79	324, 70	287, 34	465, 48	447, 57
ParaSub	51, 12	49, 11	42, 10	53, 8	54, 7	52, 9
PreSub	215, 46	201, 45	167, 38	240, 48	224, 36	226, 41
Sub	376, 52	337, 58	270, 62	416, 60	419, 45	400, 54
Tail	443, 66	422, 86	360, 77	488, 68	513, 79	485, 87
**Left side**						
Whole hippocampus	2, 927, 307	2, 652, 401	2, 182, 426	3, 233, 34	3, 295, 320	3, 058, 356
CA1	568, 73	519, 87	437, 94	596, 72	634, 74	586, 78
CA3	163, 28	149, 29	125, 28	174, 27	175, 26	161, 24
CA4	231, 27	206, 38	171, 34	251, 28	260, 32	238, 30
DG	261, 31	234, 41	194, 39	289, 33	299, 35	271, 35
Fimbria	60, 19	57, 20	39, 20	83, 15	93, 17	75, 18
Fissure	167, 29	162, 33	143, 36	155, 21	153, 32	161, 32
Hata	53, 12	49, 11	43, 10	58, 10	59, 11	54, 11
Molecular Layer	446, 60	411, 79	322, 76	289, 33	485, 59	452, 57
ParaSub	52, 11	48, 12	42, 10	52, 9	51, 8	50, 8
PreSub	246, 43	216, 43	175, 42	278, 47	257, 31	251, 43
Sub	381, 50	333, 59	271, 64	426, 54	432, 46	407, 48
Tail	443, 66	429, 82	361, 74	539, 75	550, 79	513, 97

*AD, Alzheimer's disease dementia; BD, bipolar disorders; HC, healthy controls; HCDELCODE,healthy controls DELCODE; HCAFFDIS, healthy controls AFFDIS; MCI, mild cognitive impairment; MDD, major depressive disorder; na, not available; SCD, subjective cognitive decline; y, years; Mean ± standard deviation*.

### Hippocampal Subfield Volumes in Cognitive Decline Groups

Considering the hippocampus, aMCI and AD (but not SCD) groups presented significantly smaller volumes bilaterally in comparison to HC (*post-hoc* tests: *p* < 0.05, Figure 2A). Moreover, in aMCI and AD groups, but not in SCD group, we detected lower volumes in left CA1, left CA4, left DG, left tail, left PreSub, and bilateral Sub when compared to HC (LSD *post-hoc* test: *p* < 0.05, Figures 2B,C). In the right CA1, right CA4, right DG, right tail, right PreSub, bilateral CA3, bilateral ParaSub, bilateral fimbria, and bilateral fissure regions (Figures 2B,C) we found no volume differences in HippSub in aMCI and SCD groups compared to HC.

In additional subgroup analyses, we investigated subjects presenting neuropathological abnormalities typical of AD. Concerning those DELCODE patients, for 6/32 (19%) patients with SCD, 20/63 (38%) with aMCI, and 16/42 (38%) patients with AD, their CSF pathology suggests AD. When we compared subgroups with a positive AD pathology to those without, we detected no significant between-group differences in HippSub (all *p* > 0.05, data not shown).

### Hippocampal Subfield Volumes in Affective Disorder Groups

No significant differences were detected on hippocampal and HippSub volumes when we compared MDD and BD groups to HC (*p* > 0.05).

### Hippocampal-Subfield Volumes and Duration of Depression

**Figure 3:**
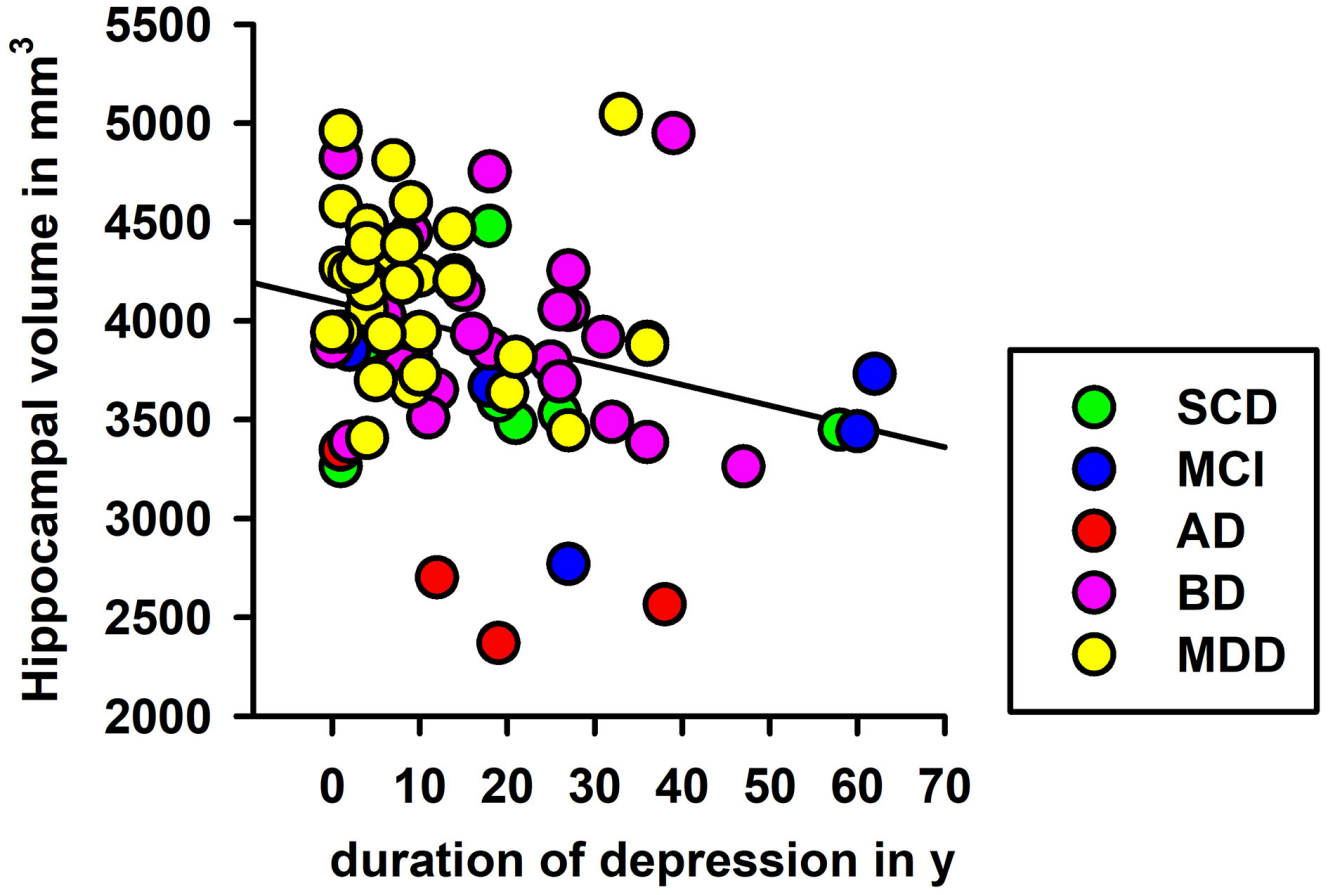
Linear regression of depression duration and left hippocampal volumes. Significant regression analyses of depression duration and left hippocampal volume are shown. AD, Alzheimer's disease; BD, bipolar disorder; L, left; aMCI, amnestic mild cognitive impairment; MDD, major depressive disorder; SCD, subjective cognitive decline; y, years.

To explore the role duration plays in years since depression onset on hippocampus volume in each hemisphere, we conducted a linear regression analysis, and noted that left, but not right hippocampal volume was significantly associated with time since first depressive episode (left hippocampus: *F* = 6.5, *p* < 0.05; Figure 3). We explored this effect further in HippSub volumes, and observed no relevant association with the time since first depressive episode and the left Sub, left CA1, left PreSub, left DG, left CA4, left fimbria, right tail, and right fimbria.

## Discussion

The main findings of our investigation are that using MRI data, hippocampal and specific HippSub volumes differed between major cognitive decline due to possible AD and early-onset of unipolar and bipolar disorders. Smaller hippocampus and most HippSub volumes were detected almost exclusively in aMCI and AD groups, while SCD, BD, and MDD groups revealed no significant smaller volumes in relation to HC. Early markers of possible neurodegeneration can therefore be seen predominantly in the left CA1, CA4, DG, tail, PreSub, and bilateral Sub regions, since significant smaller volumes were found in aMCI and ADD groups, but not in early-onset mood disorders (MDD, BD). Of note, the duration in years since first depressive episode was significantly related to the volume of left hippocampus in all patient groups. Based on the present study, the HippSub right CA1, CA4, DG, tail, PreSub, and bilateral CA3, ParaSub, fimbria, and fissure regions seem more resilient against neurodegeneration in aMCI and SCD patients. These findings may partially reflect the existing variability at certain stages of cognitive decline, as other studies have already demonstrated a volume decrease in MCI patients (Zhao et al., 2019).

A unique finding in this investigation was the significant difference seen between aMCI and BD in the left PreSub region, which could function as a suitable imaging marker. If replicated, smaller volumes in the left PreSub might prove to be the earliest indications of hippocampal-volume differences due to cognitive impairment distinct from those with bipolar mood disorders. There is evidence that both ß-amyloid and tau pathology assessed via CSF are relevant factors in lower HippSub volumes due to AD's cognitive spectrum (Tardif et al., 2018; Filho et al., 2021). As we failed to detect significant volume differences in patients with cognitive impairment with and without AD-typical CSF pathology, there might be additional mechanisms contributing to HippSub decline in our patients. Nevertheless, we could not exclude the possibility of insufficient power to detect differences, considering the relatively few subgroup samples. Further studies are needed with larger patient cohorts to differentiate the proposed underlying mechanisms of AD in HippSub volume loss. The aforementioned literature suggests other mechanisms of HippSub volume degeneration in the AD spectrum such as genes, iron accumulation, or even neuroprotective factors (Foo et al., 2020; Foster et al., 2020; Wang et al., 2020). Some of these factors may be partly responsible for the PreSub volume loss in AD, aMCI vs BD patients that we detected.

Overall, we identified neither smaller hippocampal nor HippSub volumes in early-onset mood disorder groups. That may be attributable both groups' similar age and similar severity of depressive symptoms. Furthermore, another explanation for no relevant differences in HippSub in mood disorder groups might be that structural differences between MDD-BD patients are likely less evident in the hippocampus or HippSub than in other brain regions such as thalamus, dorsolateral, and medial prefrontal cortex as well as parietal regions (Schmaal et al., 2020). The lack of smaller HippSub volumes in MDD and BD might be due to the fact that the AFFDIS cohort recruited patients undergoing antidepressant therapy. As shown lately in a survey by Han et al. (2016), drug-naïve MDD patients revealed a pattern of smaller volumes in Sub, CA2-4, DG in comparison to healthy controls. On the other hand, other factors such as early-life stress, or rs1360780 polymorphism of the FKBP5 gene (referring to the hypothalamic-pituitary-adrenal axis) associated with some smaller HippSub volumes (Mikolas et al., 2019) might also have enabled variation in our sample (data not available). Genetic architecture with different genetic loci (Hibar et al., 2017) could have a major influence on disease-specific HippSub volumes, which might explain the absence of HippSub volume reduction observed in groups with mood disorders. In contrast to our findings, BD patients have also demonstrated reduced hippocampal CA1, GCL volumes (Han et al., 2019). Smaller volumes have been observed in the PreSub and Sub regions in a subgroup of BD patients (Janiri et al., 2019), an evidence to which our left PreSub findings, in contrast to aMCI, appear to be in line with. One factor that might explain why our BD patients revealed no major hippocampal volume reductions is that, differently from ours, their cohort was heterogeneous, and not characterized by a predominantly depressive subtype. Our results, however, support the findings from a recent investigation showing no smaller volumes in MDD patients via high-resolution 7-Tesla MRI (Tannous et al., 2020). As in this study only HippSub volumes and not shape alterations were assessed, therefore we cannot identify if HippSub deformations coinciding with unaltered volumes were seen, as has been reported in MDD (Ballmaier et al., 2008; Cole et al., 2010).

Our findings suggest that depression's duration has a significant impact on left hippocampal volume, indicating that the time since first depressive episode plays an important role in hippocampal degeneration. This concurs with the knowledge that lower hippocampal volumes are associated with a poorer clinical outcome and more depressive episodes (Videbech and Ravnkilde, 2004; MacQueen and Frodl, 2011). However, when further exploring specific HippSub volumes, we observed no relationship between the duration since first depressive episode and HippSub volumes. Further studies with larger cohorts should be conducted to identify whether the duration since depressive manifestation affects HippSub volumes in a more relevant manner.

The limitations of our study concern the sample size of groups and subgroups, restricting additional conclusions in terms of clinical representation, applicability and neurobiological foundations. For instance, cognitive assessments comparable to DELCODE were not available in the AFFDIS cohort, with which we could have additionally investigated whether cognitive impairment across disorders relate to hippocampus or HippSub volume decline. A further potential limitation is the age difference between groups in both cohorts, with younger patients in the AFFDIS than the DELCODE cohort. Our analyses were controlled for age and eTIV (as covariates), but it would have been interesting to see if differences across patient groups would indeed hold when comparing older participants in mood disorder groups. Future studies addressing this aspect should also consider the potential risk of misclassifying participants with late-onset depression, since depressive episodes can be initial manifestations of neurodegeneration. However, as molecular markers have not yet been assessed in patients with affective disorders or in some patients with cognitive decline and possible AD, no general conclusions about the molecular mechanisms of neurodegeneration can be drawn for our patient groups. Cognitive decline in early-onset depression is usually not clinically associated with the neurodegenerative process, and it is often less severe (Jamieson et al., 2019) and affects specific cognitive subdomains such as language, memory, and cognitive flexibility, as recently reported (Ang et al., 2020). Thus, the manifestation age of depression is clinically relevant for the pattern and severity of cognitive decline, while also being a risk factor for later cognitive decline (Brzezińska et al., 2020). The increasing grade of severity in cognitive decline observed in late-onset compared to early-onset depression age might thus be accompanied by decreasing hippocampal and HippSub volumes.

In addition, our findings comprised cross-sectional structural imaging data and not longitudinal comparisons, through which more insight into intraindividual changes in HippSub volumes can be gained. Further studies combining functional data could better elucidate the significance of neuropathological processes in the HippSub for cognitive impairment. Lastly, potential influences of the treatment history on hippocampal and HippSub volumes could not be determined in the absence of comparable information across disorders.

Our study showed that hippocampus and HippSub volumes differ between cognitive decline due to possible AD and early-onset mood disorders. The left PreSub is a structure apparently affected in aMCI and AD subjects, but not in BD patients. This sheds new light into a possible marker differentiating correlates of neurodegeneration due to minor and major cognitive decline and BD. Conversely, we detected no relevant field and subfield volume decline in BD and MDD groups. Most strikingly, we found that the time since the first depressive episode was negatively associated with left hippocampal volume in all disorder groups. This time effect is a potentially important hallmark supporting hippocampal volume reduction as a continuum extending from mood disorders, and cognitive deterioration to AD. This finding may advance the comprehension of the relationship between depression and AD. The usage of sophisticated tools, such as machine learning, in identifying multivariate patterns in much larger groups should consider this feature.

## Data Availability Statement

The raw data supporting the conclusions of this article will be made available by the authors, without undue reservation.

## Ethics Statement

The studies involving human participants were reviewed and approved by ethical committee's. The DELCODE study protocol was coordinated by the ethical committee of the medical faculty of the University of Bonn and approved by all participating sites ethical committees [Berlin (Charité, University Medicine), Bonn, Cologne, Göttingen, Magdeburg, Munich (Ludwig-Maximilians-University), Rostock, and Tübingen]. The AFFDIS study protocol was approved by the ethical committee of the medical faculty of the University of Goettingen. The patients/participants provided their written informed consent to participate in this study. Written informed consent was obtained from the individual(s) for the publication of any potentially identifiable images or data included in this article.

## Financial Disclosure Statement

ASc got funding from Novartis, Diagnostik Netz BB (travel and speaker honoraria) and gained research support from German Federal Ministry of Research (BMBF), Actelion and Helmholtz Foundation Michael J Fox Foundation. CB received honoraria as a diagnostic consultant for Boehringer Ingelheim. DJ has obtained funding for travel from Pfizer GmbH. IK has obtained funding from the German ministry of economic cooperation and development. JP got research support for travel or speaker honoraria from Axon, CHDI, and UK DRI. He received research funding from DFG, BMBF, and UK DRI. JW has obtained research support from the Eli Lilly Advisory Board, Pfizer, MSD, and med Update GmbH (travel and speaker honoraria). He obtained research support from the BMBF. MH received funding for research support from the DFG. OP has obtained research support for travel or speaker honoraria from Schwabe. He has received funding from Eli Lilly, Lundbeck, Genentech, Biogen, Roche, Pharmatrophix, Novartis, Janssen, and Probiodrug. ST has gained support (travel or speaker honoraria) from MSD Sharp and Dohme GmbH Quality circle for physicians in Kühlungsborn and research support from ROCHE, Roche Pharma AG, Lilly Deutschland GmbH, BMBF, and Ministry of Economics of the State Mecklenburg Western Pomerania.

## Author Contributions

RG-M designed the study. NH and RG-M wrote the manuscript. ASi, NH, and RG-M analyzed the data. AC, ASi, ASc, ASp, BE-W, CB, CM, DJ, ED, ES, FB, FJ, JH, JP, JW, KB, KF, KS, LD, MM, MT, MH, NR, OP, PD, RG-M, RV, and ST contributed to data collection. All authors critically revised the manuscript. All authors made significant intellectual contributions, reviewed, and accepted this work before submission.

## Conflict of Interest

The authors declare that the research was conducted in the absence of any commercial or financial relationships that could be construed as a potential conflict of interest.

## References

[B1] Ang Y. S. Frontero N. Belleau E. Pizzagalli D. A. (2020). Disentangling vulnerability, state and trait features of neurocognitive impairments in depression. Brain 143, 3865–3877. 10.1093/brain/awaa314 33176359 PMC7805803

[B2] Ballmaier M. Narr K. L. Toga A. W. Elderkin-Thompson V. Thompson P. M. Hamilton L. (2008). Hippocampal morphology and distinguishing late-onset from early-onset elderly depression. Am. J. Psychiatry. 165, 229–237. 10.1176/appi.ajp.2007.07030506 17986679 PMC2834288

[B3] Berridge M. J. (2013). Dysregulation of neural calcium signaling in Alzheimer disease, bipolar disorder and schizophrenia. Prion 7, 2–13. 10.4161/pri.21767 22895098 PMC3609045

[B4] Brown E. M. Pierce M. E. Clark D. C. Fischl B. R. Iglesias J. E. Milberg W. P.. (2020). Test-retest reliability of FreeSurfer automated hippocampal subfield segmentation within and across scanners Neuroimage 210:116563. 10.1016/j.neuroimage.2020.116563 31972281

[B5] Brzezińska A. Bourke J. Rivera-Hernández R Tsolaki M. Wozniak J. Kazmierski J. (2020). Depression in dementia or dementia in depression? systematic review of studies and hypotheses. Curr. Alzheimer Res. (2020) 17, 16–28. 10.2174/1567205017666200217104114 32065103

[B6] Cao B. Passos I. C. Mwangi B. Amaral-Silva H. Tannous J. Wu M. J. (2017). Hippocampal subfield volumes in mood disorders. Mol. Psychiatry 22, 1352–1358. 10.1038/mp.2016.262 28115740 PMC5524625

[B7] Carlesimo G. A. Piras F. Orfei M. D. Iorio M. Caltagirone C. Spalletta G. (2015). Atrophy of presubiculum and subiculum is the earliest hippocampal anatomical marker of Alzheimer's disease. Alzheimers Dement (Amst). 1, 24–32. 10.1016/j.dadm.2014.12.001 27239489 PMC4876901

[B8] Cole J. Toga A. W. Hojatkashani C. Thompson P. Costafreda S. G. Cleare A. J.. (2010). Subregional hippocampal deformations in major depressive disorder. J. Affect. Disord. 126, 272–277. 10.1016/j.jad.2010.03.004 20392498 PMC3197834

[B9] Dale A. M. Fischl B. Sereno M. I. (1999). Cortical surface-based analysis. I. Segmentation and surface reconstruction. Neuroimage 9, 179–194. 10.1006/nimg.1998.0395 9931268

[B10] de Flores R. La Joie R. Chételat G. (2015). Structural imaging of hippocampal subfields in healthy aging and Alzheimer's disease. Neuroscience 309, 29–50. 10.1016/j.neuroscience.2015.08.033 26306871

[B11] Donovan N. J. Locascio J. J. Marshall G. A. Gatchel J. Hanseeuw B. J. Rentz D. M.. (2018). Harvard aging brain study: longitudinal association of amyloid beta and anxious-depressive symptoms in cognitively normal older adults. Am. J. Psychiatry 175, 530–537. 10.1176/appi.ajp.2017.17040442 29325447 PMC5988933

[B12] Elvsåshagen T. Zuzarte P. Westlye L. T. Bøen E. Josefsen D. Boye B.. (2016). Dentate gyrus-cornu ammonis (CA) 4 volume is decreased and associated with depressive episodes and lipid peroxidation in bipolar II disorder: Longitudinal and cross-sectional analyses. Bipolar Disord. 18, 657–668. 10.1111/bdi.12457 27995733

[B13] Enache D. Winblad B. Aarsland D. (2011). Depression in dementia: epidemiology, mechanisms, and treatmen. Curr. Opin. Psychiatry 24, 461–472. 10.1097/YCO.0b013e32834bb9d4 21926624

[B14] Filho G. B. de Souza Duran F. L. Squarzoni P. Coutinho N. A. M. Rosa P. G. P. Torralbo L.. (2021). Hippocampal subregional volume changes in elders classified using positron emission tomography-based Alzheimer's biomarkers of β-amyloid deposition and neurodegeneration. J. Neurosci. Res. 99, 481–501. 10.1002/jnr.24739 33073383

[B15] Fischl B. Sereno M. I. Dale A. M. (1999). Cortical surface-based analysis. II: Inflation, flattening, and a surface-based coordinate system. Neuroimage 9, 195–207. 10.1006/nimg.1998.0396 9931269

[B16] Foo H. Thalamuthu A. Jiang J Koch F Mather K. A. Wen W. Sachdev P. S. (2020). Associations between Alzheimer's disease polygenic risk scores and hippocampal subfield volumes in 17,161 UK Biobank participants. Neurobiol. Aging 98, 108–115. 10.1016/j.neurobiolaging.2020.11.002 33259984

[B17] Foster C. M. Kennedy K. M. Daugherty A. M. Rodrigue K. M. (2020). Contribution of iron and Abeta to age differences in entorhinal and hippocampal subfield volume. Neurology 95, e2586–e2594. 10.1212/WNL.0000000000010868 32938781 PMC7682827

[B18] Garimella A. Rajguru S. Singla U. K. Alluri V. (2020). Marijuana and the hippocampus: a longitudinal study on the effects of marijuana on hippocampal subfields. Prog. Neuropsychopharmacol. Biol. Psychiatry 101:109897. 10.1016/j.pnpbp.2020.109897 32119881

[B19] Genzel L. Dresler M. Cornu M. Jäger E. Konrad B. Adamczyk M.. (2015). Medial prefrontal-hippocampal connectivity and motor memory consolidation in depression and schizophrenia. Biol. Psychiatry 77, 177–186. 10.1016/j.biopsych.2014.06.004 25037555

[B20] Han K. M. Kim A. Kang W. Kang Y. Kang J. Won E.. (2019). Hippocampal subfield volumes in major depressive disorder and bipolar disorder. Eur. Psychiatry 57, 70–77. 10.1016/j.eurpsy.2019.01.016 30721801

[B21] Han K. M. Won E. Sim Y. Tae W. S. (2016). Hippocampal subfield analysis in medication-naïve female patients with major depressive disorder. J. Affect. Disord. 194, 21–29. 10.1016/j.jad.2016.01.019 26802503

[B22] Hanseeuw B. J. Van Leemput K. Kavec M. Grandin C. Seron X. Ivanoiu A. (2011). Mild cognitive impairment: differential atrophy in the hippocampal subfields. AJNR Am. J. Neuroradiol. 32, 1658–1661. 10.3174/ajnr.A2589 21835940 PMC3268157

[B23] Haukvik U. K. Gurholt T. P. Nerland S. Elvsåshagen T. Akudjedu T. N. Alda M.. (2020). *In vivo* hippocampal subfield volumes in bipolar disorder-A mega-analysis from the enhancing neuro imaging genetics through meta-analysis Bipolar Disorder Working Group. Hum Brain Mapp. (2020). 10.1002/hbm.25249. [Epub ahead of print]. 33073925 PMC8675404

[B24] Heser K. Tebarth F. Wiese B. Eisele M. Bickel H. Köhler M.. (2013). Age of major depression onset, depressive symptoms, and risk for subsequent dementia: results of the German study on Ageing, Cognition, and Dementia in Primary Care Patients (AgeCoDe). Psychol. Med. 43, 1597–1610. 10.1017/S0033291712002449 23137390

[B25] Hibar D. P. Adams H. H. H. Jahanshad N. Chauhan G. Stein J. L. Hofer E.. (2017). Novel genetic loci associated with hippocampal volume. Nat. Commun. 8:13624. 10.1038/ncomms13624 28098162 PMC5253632

[B26] Iglesias J. E. Augustinack J. C. Nguyen K. Player C. M. Player A. Wright M.. (2015). A computational atlas of the hippocampal formation using *ex vivo*, ultra-high resolution MRI: Application to adaptive segmentation of *in vivo* MRI. Neuroimage 115, 117–137. 10.1016/j.neuroimage.2015.04.042 25936807 PMC4461537

[B27] Jack C. R. Jr. Bennett D. A. Blennow K. Carrillo M. C. Dunn B. Haeberlein S. B.. (2018). NIA-AA Research Framework: toward a biological definition of Alzheimer's disease. Alzheimers Dement. 14, 535–562. 10.1016/j.jalz.2018.02.018 29653606 PMC5958625

[B28] Jacobs H. I. L. Augustinack J. C. Schultz A. P. Hanseeuw B. J. Locascio J. Amariglio R. E.. (2020). The presubiculum links incipient amyloid and tau pathology to memory function in older persons. Neurology 94, e1916–e1928. 10.1212/WNL.0000000000009362 32273431 PMC7274925

[B29] Jamieson A. Goodwill A. M. Termine M. Campbell S. Szoeke C. (2019). Depression related cerebral pathology and its relationship with cognitive functioning: a systematic review. J. Affect. Disord. 250, 410–418. 10.1016/j.jad.2019.03.042 30878653

[B30] Janiri D. Sani G. De Rossi P. Piras F. Banaj N. Ciullo V.. (2019). Hippocampal subfield volumes and childhood trauma in bipolar disorders. J. Affect. Disord. 253, 35–43. 10.1016/j.jad.2019.04.071 31022627

[B31] Jessen F. Amariglio R. E. Buckley R. F. van der Flier W. M. Han Y. Molinuevo J. L.. (2020). The characterisation of subjective cognitive decline. Lancet Neurol. 19, 271–278. 10.1016/S1474-4422(19)30368-0 31958406 PMC7062546

[B32] Jessen F. Amariglio R. E. van Boxtel M. Breteler M. Ceccaldi M. Chételat G.. (2014). Subjective Cognitive Decline Initiative (SCD-I) Working Group: a conceptual framework for research on subjective cognitive decline in preclinical Alzheimer's disease. Alzheimers Dement. 10, 844–852. 10.1016/j.jalz.2014.01.001 24798886 PMC4317324

[B33] Jessen F. Spottke A. Boecker H. Brosseron F. Buerger K. Catak C.. (2018). Design and first baseline data of the DZNE multicenter observational study on predementia Alzheimer's disease (DELCODE). Alzheimers Res. Ther. 10:15. 10.1186/s13195-017-0314-2 29415768 PMC5802096

[B34] La Joie R. Perrotin A. de La Sayette V. Egret S. Doeuvre L. Belliard S.. (2013). Hippocampal subfield volumetry in mild cognitive impairment, Alzheimer's disease and semantic dementia. Neuroimage Clin. 14, 155–162. 10.1016/j.nicl.2013.08.007 24179859 PMC3791274

[B35] MacQueen G. Frodl T. (2011). The hippocampus in major depression: evidence for the convergence of the bench and bedside in psychiatric research? Mol. Psychiatry (2011) 16, 252–264. 10.1038/mp.2010.80 20661246

[B36] McKhann G. M. Knopman D. S. Chertkow H. Hyman B. T. Jack C. R. Jr Kawas C. H.. (2011). The diagnosis of dementia due to Alzheimer's disease: recommendations from the National Institute on Aging-Alzheimer's Association workgroups on diagnostic guidelines for Alzheimer's disease. Alzheimers Dement. 7, 263–269. 10.1016/j.jalz.2011.03.005 21514250 PMC3312024

[B37] Mikolas P. Tozzi L. Doolin K. Farrell C. O'Keane V. Frodl T. (2019). Effects of early life adversity and FKBP5 genotype on hippocampal subfields volume in major depression. J. Affect. Disord. 252, 152–159. 10.1016/j.jad.2019.04.054 30986730

[B38] Rizzolo L. Narbutas J. Van Egroo M. Chylinski D. Besson G. Baillet M. (2021). Relationship between brain AD biomarkers and episodic memory performance in healthy aging. Brain Cogn. 148:105680. 10.1016/j.bandc.2020.105680 33418512

[B39] Schmaal L. Pozzi E. Ho T. C. van Velzen L. S. Veer I. M. Opel N.. (2020). ENIGMA MDD: seven years of global neuroimaging studies of major depression through worldwide data sharing. Transl. Psych. 10:172. 10.1038/s41398-020-0842-6 32472038 PMC7260219

[B40] Schmaal L. Veltman D. J. van Erp T. G. Sämann P. G. Frodl T. Jahanshad N.. (2016). Subcortical brain alterations in major depressive disorder: findings from the ENIGMA Major Depressive Disorder working group. Mol. Psychiatry 21, 806–812. 10.1038/mp.2015.69 26122586 PMC4879183

[B41] Singh-Manoux A. Dugravot A. Fournier A. Abell J. Ebmeier K. Kivimäki M.. (2017). Trajectories of depressive symptoms before diagnosis of dementia: a 28-year follow-up study. JAMA Psychiatry 74, 712–718. 10.1001/jamapsychiatry.2017.0660 28514478 PMC5710246

[B42] Szymkowicz S. M. McLaren M. E. O'Shea A. Woods A. J. Anton S. D. Dotson V. M. (2017). Depressive symptoms modify age effects on hippocampal subfields in older adults. Geriatr. Gerontol. Int. 17, 1494–1500. 10.1111/ggi.12901 27696657 PMC5376518

[B43] Tannous J. Godlewska B. R. Tirumalaraju V. Soares J. C. Cowen P. J. Selvaraj S. (2020). Stress, inflammation and hippocampal subfields in depression: a 7 Tesla MRI Study. Transl. Psychiatry 10:78. 10.1038/s41398-020-0759-0 32098947 PMC7042360

[B44] Tardif C. L. Devenyi G. A. Amaral R. S. C. Pelleieux S. Poirier J. Rosa-Neto P.. (2018). Regionally specific changes in hippocampal circuity accompany progression of cerebrospinal fluid biomarkers in preclinical Alzheimer's disease. Hum. Brain Mapp. 39, 971–984. 10.1002/hbm.23897 29164798 PMC6866392

[B45] Treadway M. T. Waskom M. L. Dillon D. G. Holmes A. J. Park M. T. M. Chakravarty M. M.. (2015). Illness progression, recent stress, and morphometry of hippocampal subfields and medial prefrontal cortex in major depression. Biol. Psychiatry 77, 285–294. 10.1016/j.biopsych.2014.06.018 25109665 PMC4277904

[B46] Videbech P. Ravnkilde B. (2004). Hippocampal volume and depression: a meta-analysis of MRI studies. Am. J. Psychiatry 161, 1957–1966. 10.1176/appi.ajp.161.11.1957 15514393

[B47] Wang S. Y. Xue X. Duan R. Gong P. Y. Jiang T. Zhang Y. D.. (2020). A TREML2 missense variant influences specific hippocampal subfield volumes in cognitively normal elderly subjects. Brain Behav. 10:e01573. 10.1002/brb3.1573 32073739 PMC7177563

[B48] Weissman D. G. Lambert H. K. Rodman A. M. Peverill M. Sheridan M. A. McLaughlin K. A. (2020). Reduced hippocampal and amygdala volume as a mechanism underlying stress sensitization to depression following childhood trauma. Depress Anxiety. 37, 916–925. 10.1002/da.23062 32579793 PMC7484449

[B49] Whelan C. D. Hibar D. P. van Velzen L. S. Zannas A. S. Carrillo-Roa T. McMahon K.. (2016). Heritability and reliability of automatically segmented human hippocampal formation subregions. Neuroimage 128, 125–137. 10.1016/j.neuroimage.2015.12.039 26747746 PMC4883013

[B50] Xu J. Tang Y. Baro C. C. Zhang X. Meng Z. Li Y. (2018). Left fimbria atrophy is associated with hippocampal metabolism in female major depressive disorder patients. Annu. Int. Conf. IEEE Eng. Med. Biol. Soc. 2018, 1136–1139. 10.1109/EMBC.2018.8512472 30440590

[B51] Xu R. Hu X. Jiang X. Zhang Y. Wang J. Zeng X. (2020). Longitudinal volume changes of hippocampal subfields and cognitive decline in Parkinson's disease. Quant. Imaging Med. Surg. 10, 220–232. 10.21037/qims.2019.10.17 31956544 PMC6960434

[B52] Yuan M. Rubin-Falcone H. Lin X. Rizk M. M. Miller J. M. Sublette M. E.. (2020). Smaller left hippocampal subfield CA1 volume is associated with reported childhood physical and/or sexual abuse in major depression: a pilot study. J. Affect. Disord. 272, 348–354. 10.1016/j.jad.2020.03.169 32553377 PMC13108372

[B53] Zhao W. Wang X. Yin C. He M. Li S. Han Y. (2019). Trajectories of the hippocampal subfields atrophy in the Alzheimer's disease: a structural imaging study. Front. Neuroinform. 13:1. 10.3389/fninf.2019.00013 30983985 PMC6450438

